# Treatment and Management of Sexual Disinhibition in Elderly Patients With Neurocognitive Disorders

**DOI:** 10.7759/cureus.18463

**Published:** 2021-10-03

**Authors:** Ashish Sarangi, Hannah Jones, Fariha Bangash, Jayasudha Gude

**Affiliations:** 1 Psychiatry, Baylor College of Medicine, Houston, USA; 2 Psychiatry, Texas Tech University Health Sciences Center, Lubbock, USA; 3 Psychiatry, Khyber Medical University, Peshawar, PAK; 4 Psychiatry, Northwell Health, New York City, USA

**Keywords:** elderly population, behavioral disturbance, geriatric psychiatry, alzheimer’s dementia, hypersexuality

## Abstract

Sexual disinhibition is uncommon but challenging symptom to address in elderly patients with neurocognitive disorders. Due to the lack of large-scale studies, there is no gold standard treatment for sexual disinhibition, and treatment is largely left up to the discretion of the provider based on the severity and onset of the patient’s symptoms. A review was conducted to investigate the non-pharmacological and pharmacological interventions for treating this condition. Articles that discussed treatments were screened for the type of treatment and possible side effects of medication if applicable. Thorough patient history should be taken prior to starting any drug therapy to rule out possible behavioral changes due to an existing medication side effect, delirium, or past mental or sexual health history. Non-pharmacological treatment has been generally recommended as first-line therapy over pharmacological treatment. Distraction/diversion of the patient when inappropriate sexual behaviors occur was the most common non-pharmacological intervention. Antidepressants were generally recommended as the first line of pharmacological treatment after attempting all possible non-pharmacological interventions. Several other categories of interventions are discussed as well in addition to the ethical implications of treating a patient for this condition.

## Introduction and background

Sexual disinhibition in patients with neurocognitive disorders presents a challenging dilemma for patients, caretakers, and healthcare workers. While the most common change in sexual behaviors with the onset of a neurocognitive disorder is a decline in sexual interest, the emergence of sexual disinhibition or sexually inappropriate behaviors has been reported in between 1.8% and 25.9% of samples of patients with neurocognitive disorders [1-3]. While much of the prior literature on this topic uses the terms “sexually inappropriate behaviors,” “sexual disinhibition” and “hypersexuality” interchangeably, more recent literature redefines this pathology in patients with neurocognitive disorders as sexual disinhibition [3,4]. This behavior can be broken down into five domains: inability to inhibit, oversharing, inappropriate comments, inappropriate exposure, and overly flirtatious [5]. Three commonly cited types of sexually inappropriate behavior include 1) sexual language that is different from the patient’s premorbid personality; 2) implied sexual acts including reading pornographic material or requesting genital care in the absence of need; and 3) overt sexual acts including groping, grabbing, public masturbating or exposing oneself to caretakers or family members [6-8].

This issue is distressing to both the patient and the caregiver. A study found that caregivers of patients exhibiting sexual disinhibition reported higher levels of caregiver burden and were more likely to want to institutionalize their patients [5]. Within the residential healthcare setting, patients with neurocognitive disorders exhibiting sexual disinhibition present a difficult challenge for healthcare workers who must prevent circumstances that could result in elder abuse while protecting the dignity and autonomy of patients.

Limited research exists on sexual disinhibition in patients with neurocognitive disorders. Due to a lack of consistency in criteria for diagnosis and wide variation in presenting symptomatic behaviors, establishing a standard for treatment will require future research. The purpose of this review is to discuss sexual disinhibition as it relates to different subsets of neurocognitive disorders along with available pharmacologic and non-pharmacologic treatments which have shown promise in treating this pathology.

Frontotemporal dementia and sexual disinhibition 

Behavioral variant frontotemporal dementia (bvFTD) is a clinical syndrome characterized by the onset of changes in behavior in concurrence with degeneration of frontal and temporal regions of the brain [9]. Most patients diagnosed with bvFTD experience a decrease in sexual behaviors including decreased affection, initiation of sexual advances, and frequency of sexual relations [1]. However, sexual disinhibition and hypersexual behaviors still occur within this population. In a study comparing sexual behaviors in patients with different subsets of neurocognitive disorders, researchers found that 13% (n=47) of bvFTD patients showed hypersexual behaviors including general disinhibition, poor impulse control, and actively seeking sexual stimulation [10]. This is expected with the general role of the frontal lobe in impulsive behaviors, making judgments, and controlling aspects of sexual behavior. One study also found an association between inappropriate sexual behaviors in a geriatric population and right frontal lobe stroke [11].

Frontal or temporal lobe lesions caused by trauma or organic brain disease such as bvFTD are the most common cause of sexual disinhibition in these patients [7,12]. Several social factors have been proposed as contributing factors as well. These include lack of a usual sexual partner, an under-stimulating or unfamiliar environment, misinterpretation of social cues, and lack of privacy [12].

Alzheimer’s disease (AD) and sexual disinhibition

There is limited research available on AD and hypersexual behaviors. One study found that in a sample of 41 patients with sexual disinhibition and neurocognitive disorders, 22% had a diagnosis of AD [2]. Many AD patients suffer from behavioral disturbances due in part to a deficiency in GABA [13]. Some case reports have seen success in treating AD patients with this pathology along with general agitation with gabapentin (200-1,200mg) [14].

Parkinson’s disease (PD) and sexual disinhibition

Much of the literature on sexual disinhibition and neurocognitive disorders consists of studies on PD patients exhibiting this behavior. Impulse control disorders (ICD) are common in patients with PD [15]. This can include compulsive gambling, shopping, eating, and hypersexual behaviors [15]. Hypersexual behaviors have been shown to be one of the earliest signs of development of ICD in PD and an estimated prevalence of 3.5% of PD patients have been shown to exhibit hypersexual behaviors [16]. Younger onset PD, male sex, and a history of behavioral problems were commonly associated with hypersexual behaviors [16,17]. Additionally, several medications used to treat PD have been linked to the development and/or exacerbation of ICD and hypersexual behaviors in these patients. One study found that 98.1% of patients (n=3,090) with ICD and PD were taking dopamine agonists or high doses of levodopa and they had an increased risk of developing hypersexual behaviors ranging from 1.7% to 3.5% [18]. Individually, dopamine agonists and levodopa have been shown to increase hypersexual behaviors in PD patients with a prevalence of 7.4% and 2.7%, respectively [17]. Therefore, it is important to consider medication regimens when assessing how best to treat sexual disinhibition in PD patients.

In patients displaying these behaviors while taking dopamine agonists, it is recommended to first try decreasing the dosage [19]. If hypersexual behaviors continue some researchers recommend switching from a dopamine agonist to levodopa [19]. A thorough patient interview including prior medical, psychological and sexual history should be conducted before starting a PD patient on a medication regimen. Additionally, patients should be assessed for a history of sexual aggression.

Assessment

As the root cause of sexual disinhibition can vary widely based on a subset of diagnoses of neurocognitive disorder, a thorough patient history should be taken including medical, social, and sexual history as well as a full medication review. Several medications have been shown to increase sexual disinhibition or worsen impulsive behaviors. It is pivotal that the patient’s medications are reviewed in full and evaluated as a potential cause of hypersexual behaviors.

History of sexual aggression or behavioral problems should be discussed as well [7,17]. While there is no DSM-V diagnosis for hypersexuality, a patient may have been exhibiting hypersexual behaviors prior to their diagnosis of a neurocognitive disorder [20]. This illustrates the importance of creating a timeline of symptoms in order to accurately treat the patient. Additionally, psychiatric history should be discussed for evidence of post-traumatic stress disorder (PTSD), depression, mania, and delirium as these conditions are associated with increased incidence of hypersexuality [7,12,21]. A review of systems should be conducted to identify possible illnesses that could be causing delirium-induced hypersexuality and a Confusion Assessment Method (CAM) screening questionnaire can be used to differentiate between a neurocognitive disorder and delirium if needed [7,22]. Possible medical causes of delirium include metabolic disturbances such as acute kidney injury and electrolyte imbalances, infections such as UTI or meningitis/encephalitis, and CNS insults such as stroke or seizure [23]. With the patient’s consent, discussions with family members and support systems may be helpful in gathering information regarding how their behavior has changed over time.

Staff training

The nature of sexual disinhibition presents a difficult case for staff in nursing facilities. Due to the sensitive nature of sexual behavior, nursing staff may feel hesitant or embarrassed to report incidents. It is important that staff are properly trained in handling and reporting these behaviors to prevent physical and emotional harm to healthcare workers. The use of role-playing and scenario discussions during staff meetings will help increase confidence in responding to inappropriate sexual behaviors in patients in a professional and dignified manner [24,25]. Training is vital to ensure that staff response does not encourage or dismiss inappropriate sexual behavior.

## Review

**Figure 1:**
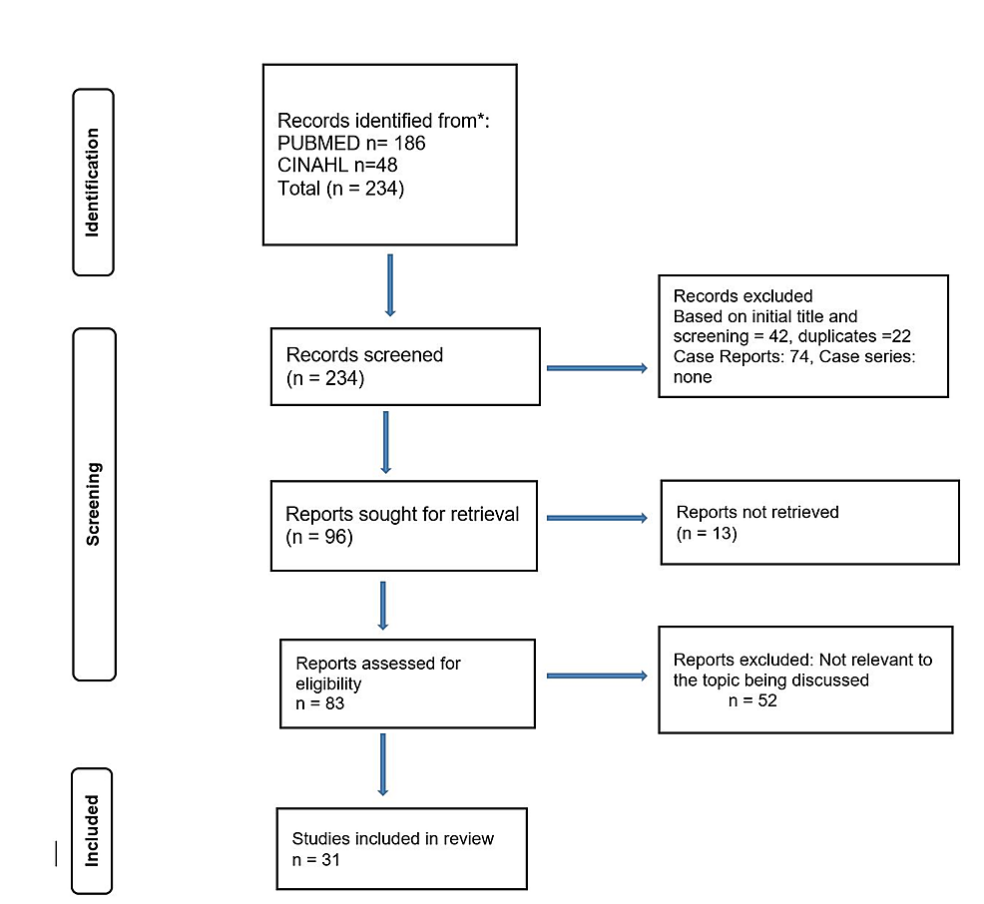
Flowchart of PubMed database search using PRISMA method

A focused literature review was conducted using the PubMed and CINAHL databases from inception till August 2021. Search terms included Sexual Disinhibition OR Hypersexuality OR Sexually inappropriate behavior AND Dementia. The search yielded a total of 234 articles. Inclusion criteria consisted of full-length, peer-reviewed papers discussing the treatment of hypersexuality/sexual disinhibition in elderly patients with any subset of dementia or neurocognitive disorder. After removing duplicate entries, letters to the editor, book chapters, and all articles that did not fit our inclusion criteria we had 31 articles to include in our review. Figure 1 demonstrates the process of our database search. Table 1 summarizes the evidence for pharmacological management of hypersexuality in patients with neurocognitive disorders. Table 2 summarizes the evidence for non-pharmacological options for management.

 

**Table 1:** Non-pharmacologic interventions for the management of hypersexuality in patients with neurocognitive disorders

Intervention	References [24, 26-37]
Removing precipitating factors	^[24,26-28]^
Distraction or diversion, redirect behavior, engage patients in activities that involve the hands, continuous activity programming in nursing homes, reducing sexual stimulation (magazines, TV)	^[26,28-34]^
Fulfill the need for intimacy/connection in other ways like having meals in groups, encourage conversation amongst peers, engage in more activities such as walking or exercise	^[35]^
Offering clothing that opens in the back	^[26,30]^
Behavioral/cognitive behavioral therapy	^[30,33,34,36]^
Sensory and environmental stimulation such as aromatherapy,​​​​​​​ music therapy, ​​​​​​​multisensory therapy, ​​​​​​​pet therapy	^[27,29,33]^
Patient and caregiver education	^[24,29,30,32,34]^
Improve patient’s self-esteem	^[37]^

 

**Table 2:** Pharmacologic interventions for the management of hypersexuality in patients with neurocognitive disorders

Medication	Mechanism of Action	Side effects	References [7,10,14,26,29-33,35,37-46]
Antidepressants: Selective serotonin reuptake inhibitors (SSRIs) (First line, first choice), Citalopram, Escitalopram, Paroxetine.	Decrease libido and may have anti obsessive effect	Insomnia, somnolence, nausea, diarrhea, headache, anorexia	^[7,10,14,26,29,30,31,33,35,37-39]^
Antidepressants: Tricyclic antidepressants (First line, second choice), Clomipramine	Decrease libido	Dry mouth, blurry vision, constipation, urine retention, recumbent tachycardia, memory impairment (Gillman)	^[31-33,38]^
Antidepressants: Trazodone	Decrease libido	Day-time sedation, orthostatic hypotension, priapism, falls and fractures, delirium	^[26,29,32,35,40]^
Antiandrogen: Cyproterone acetate (second line)	Reduction in serum testosterone level by inhibiting LH and FSH	Gynecomastia, galactorrhoea, elevated blood glucose, depression, osteoporosis,	^[7,14,26,29,31,33,37,38]^
Antiandrogen: Medroxyprogesterone acetate (second line)	Reduction in testosterone	Sedation, weight gain, hot flashes, depression, elevated blood glucose	^[7,14,26,29-33,37,38,41]^
Antiandrogen: Finesteride	Reduction in testosterone	Gynecomastia, testicular pain, depression	^[26]^
Estrogens (Third line): Estradiol, Estrone, Diethylstilbestrol	Decrease testosterone and decrease libido	Weight gain, gynecomastia, venous thromboembolism, risk of cardiovascular side effects, fluid retention, GI effects	^[7,14,26,29,31,37,38]^
GnRH and LHRH analogues: Leuprolide, Triptorelin, Goserelin	Decrease testosterone and decrease libido	Hot flashes, decreased erectile dysfunction	^[7,26,30,31,33,37,38]^
Atypical antipsychotics: Risperidone (first choice), Olanzapine (alternative first choice), Quetiapine, Aripiprazole, Clozapine	Block dopamine receptors to decrease libido	Stroke, death, pneumonia, cognitive decline, extrapyramidal symptoms, sedation, gait disturbances, falls, tardive dyskinesia, delirium, QT prolongation, increases in UTI and respiratory infections, peripheral edema Olanzapine specific: Hyperlipidemia, hyperglycemia, increased risk of Type II diabetes	^[7,10,29,31,33,35,37]^
Typical Antipsychotics: Haloperidol, Zuclopenthixol	Block dopamine receptors to decrease libido	Stroke, death, pneumonia, cognitive decline, extrapyramidal symptoms, sedation, gait disturbances, falls, tardive dyskinesia, delirium, QT prolongation, increases in UTI and respiratory infections, peripheral edema Extrapyramidal effects of Zuclopenthixol- discontinued in a case study due to this side effect	^[7,27,29,30,31,42]^
Mood stabilizer: Carbamazepine, Oxcarbazepine	Unknown may help control dementia-related disinhibition May help lower testosterone levels leading to decreased libido	Sedation, depression, Stevens–Johnson syndrome, agranulocytosis, hyponatremia	^[7,26,29,31-33,37,43]^
Mood stabilizer: Gabapentin	Increase GABA in AD patients	Sedation, depression, ataxia, tremor	^[7,14,26,31,43]^
Mood stabilizer: Valproate	Unknown	Tremor, sedation, falls, weight gain, hair loss, empathic dysfunction	^[26,29]^
Anxiolytic: Buspirone	Unknown	Dizziness, headache, nausea, nervousness, paresthesia (Politis)	^[35]^
Benzodiazepines	Unknown	Exacerbation of hypersexual behaviors	^[30]^
Beta-Blockers: Pindolol, Propranolol	Decrease adrenergic drive	Fatigue, hypotension, bradycardia, bronchospasm, depression	^[26,29,31]^
H2-receptor antagonist: Cimetidine	Antiandrogen actions	Worsening cognition, dizziness, nausea, arthralgia, headache	^[26,29,31,37,44]^
Potassium-sparing diuretic: Spironolactone	Antiandrogen actions	Hyperkalemia, gynecomastia, gastrointestinal ulcers	^[26,31,46]^
Antifungals: Ketoconazole	Antiandrogen actions	Sedation, headache, rash, photosensitivity, pruritus, hepatotoxicity, gastrointestinal upset	^[26,31,44]^
Cholinesterase inhibitors: Rivastigmine, Galantamine	Reduce behavioral symptoms by improving cognitive functioning	Nausea, urinary incontinence, syncope, the potential for the emergence of hypersexuality Conflicting evidence on benefit of this treatment	^[7,26,29,31-33,45]^
NMDA receptor antagonist: Memantine, Amantadine	Reduce behavioral symptoms by improving cognitive functioning	Conflicting evidence on benefit of this treatment	^[10,29,31,32]^
L-Tryptophan supplementation	Increases 5-HT synthesis in brain-stimulating 5-HT release and function	High blood glucose, increased risk of bladder cancer, eosinophilia-myalgia syndrome	^[46]^

A consensus on the best method for treating sexual disinhibition in elderly patients with dementia has yet to be made. The goal of treatment should be to decrease sexual behavior to an appropriate level rather than completely eradicate it [24,30,32]. Non-pharmacologic interventions should be implemented before starting the patient on medication due to the risks associated with many of the pharmacological treatments [12]. Many of the drugs used to treat sexual disinhibition may put the patient at an increased risk of more rapid cognitive decline and can carry considerable risk for adverse events [12,29]. Therefore, careful implementation of non-pharmacologic techniques should be conducted and documented before implementing drug therapy.

Most of the literature on non-pharmacological techniques recommends “distraction or diversion” of patients when inappropriate sexual behavior begins. Reminding patients of where they are and why this behavior is inappropriate may help orient patients to time and place. It has also been cited that some patients may use sexual performance as a means to increase self-esteem [37]. Patients suffering from reduced self-esteem secondary to loss of autonomy may benefit from physical therapy to increase their ability to perform day-to-day tasks [37].

If non-pharmacologic interventions fail to stop the behavior or if the patient is determined to be a direct threat to themselves or others, pharmacologic treatments can be started [29]. Due to the complex nature of sexual disinhibition and varying origins of this behavior in patients with dementia, treatment will be most successful when tailored to the patient’s specific disease presentation. There are limited studies available on the pharmacologic treatment of sexual disinhibition and larger studies are necessary to elucidate a definitive medication regimen. Additionally, there is little data available on treating this pathology in females [38]. A systematic review by Guay provides a good place to start when treating patients afflicted with AD, vascular dementia, or unspecified dementia [38]. Serotonergic agents including SSRIs and TCAs are recommended as a first-line treatment, followed by antiandrogens as a second line, and LHRH-agonists and estrogens as a third line [38]. A more recent literature review conducted by Ibrahim determined SSRIs to be the first line, antipsychotics to be the second line of treatment, and hormonal modulators to be the third line due to the cost and side effects [30].

SSRIs tended to be the most cited as the best first-line treatment for elderly patients with sexual disinhibition due to lower risks and less burdensome side effects. There were a few exceptions to this recommendation. In cases where patients present with pathologic irritability or unstable mood, some researchers have suggested using an antipsychotic medication as a first-line treatment instead of an SSRI due to the risk of exacerbation of these symptoms [36]. Additionally, patients presenting with pre-existing apathy may see exacerbated symptoms while taking SSRIs. Lastly, citalopram has been seen to be poorly tolerated in patients with Lewy body dementia (LBD) [29].

The decision to treat sexual disinhibition with antipsychotics should be made carefully. The Food and Drug Administration has not approved any antipsychotics to treat behavioral problems in dementia patients [47]. Only risperidone is approved for use in Europe for treating aggression and disruptive behaviors in patients with AD [47]. This is due to the large number of adverse events associated with use including stroke, mortality, and hallucination [47]. One study demonstrated that 77%-85% of patients treated with antipsychotic medications for a variety of conditions opted to discontinue treatment due to adverse side effects [29]. In general, atypical antipsychotics such as risperidone or olanzapine are preferred in elderly patients over typical antipsychotics such as haloperidol due to better tolerance [32]. Patients with LBD tend to be more sensitive to antipsychotics and have an increased risk of morbidity and mortality [29]. Additionally, antipsychotics tend to worsen extrapyramidal symptoms in AD patients and worsen gait disturbances in patients with PD and LBD [29]. Desai suggests using quetiapine over other atypical antipsychotics in patients who are at risk of extrapyramidal symptoms [29].

Benzodiazepines should be prescribed for sexual disinhibition with caution as well. Some studies cite an increase in hypersexual behaviors with both administration and withdrawal of medication [30]. Additionally, benzodiazepines tend to be poorly tolerated due to side effects [35].

The use of cimetidine as a non-hormonal anti-androgen treatment was most cited from an article by Wiseman who conducted a small study (n=20) on elderly patients exhibiting hypersexual behaviors with dementia. This study found that 14 patients' hypersexual behaviors improved with cimetidine treatment alone while six patients improved with a combination treatment of cimetidine administered in conjunction with ketoconazole or spironolactone [44].

The use of cholinesterase inhibitors to treat sexual disinhibition presents conflicting evidence. Rivastigmine and donepezil are both cholinesterase inhibitors; however, rivastigmine has been shown to help many patients with sexual disinhibition while donepezil has been shown to exacerbate these symptoms [31,45,48,49]. More research is needed in order to determine the benefit of this class of medications for treating sexual disinhibition.

It is essential to discuss the ethical implications of treating sexual disinhibition in patients with dementia. Restricting a patient’s sexual expression without their consent through the use of “chemical castration” -- i.e., medication, particularly estrogen therapies -- can be considered a human rights violation. Cognitive impairment adds a complex factor to treating this condition as the patient is unable to consent to treatment or speak to how they wish their behavior will be modified. Additionally, while most cognitively impaired patients displaying sexual disinhibition do not face legal consequences, they could suffer other social consequences such as loss of relationships or denial from housing at residential nursing homes. Consent from a legal representative who can speak to what the patient would have wanted prior to their cognitive decline is essential yet complex as well. Allowing the legal representative, who is often the patient’s caregiver, to consent to treatment presents another opportunity for unjust treatment of the patient as some forms of sexual disinhibition may not be particularly harmful to the patient while being deeply disruptive to the caregiver.

## Conclusions

While sexual disinhibition can be an uncomfortable issue to address, it is important to prioritize the physical and mental health of the patient and those who may be impacted by the hypersexual acts. The medical provider must evaluate cases of sexual disinhibition in each patient carefully and only recommend pharmacological treatment when deemed necessary. Unwavering consistency and support must be present from the entire care team to ensure the patient is treated appropriately and with the utmost respect.
